# Selective decontamination of the digestive tract in colorectal surgery reduces anastomotic leakage and costs: a propensity score analysis

**DOI:** 10.1007/s00423-022-02540-6

**Published:** 2022-05-13

**Authors:** Andreas Bogner, Maximilian Stracke, Ulrich Bork, Steffen Wolk, Mathieu Pecqueux, Sandra Kaden, Marius Distler, Christoph Kahlert, Jürgen Weitz, Thilo Welsch, Johannes Fritzmann

**Affiliations:** 1 Department of Visceral, Thoracic and Vascular Surgery, University Hospital and Faculty of Medicine Carl Gustav Carus, Technische Universität Dresden, Dresden, Germany; 2 National Center for Tumor Diseases (NCT/UCC), Dresden, Germany; 3 German Cancer Research Center (DKFZ), Heidelberg, Germany; 4 Faculty of Medicine and University Hospital Carl Gustav Carus, Technische Universität Dresden, Dresden, Germany; 5 Helmholtz-Zentrum Dresden-Rossendorf (HZDR), Dresden, Germany; 6 Department of Gastrointestinal, Thoracic and Vascular Surgery, Faculty of Medicine Carl Gustav Carus, Technische Universität Dresden, Dresden, Germany; 7 Clinical Pharmacy, University Hospital and Faculty of Medicine Carl Gustav Carus, Technische Universität Dresden, Dresden, Germany; 8 Department of General, Visceral and Thoracic Surgery, Oberschwabenklinik Ravensburg, Ravensburg, Germany

**Keywords:** Oral antibiotics, Mechanical bowel preparation, Anastomotic leakage, Colorectal surgery, Surgical site infection, Cost reduction

## Abstract

**Purpose:**

Anastomotic leakage (AL) and surgical site infection (SSI) account for most postoperative complications in colorectal surgery. The aim of this retrospective trial was to investigate whether perioperative selective decontamination of the digestive tract (SDD) reduces these complications and to provide a cost-effectiveness model for elective colorectal surgery.

**Methods:**

All patients operated between November 2016 and March 2020 were included in our analysis. Patients in the primary cohort (PC) received SDD and those in the historical control cohort (CC) did not receive SDD. In the case of rectal/sigmoid resection, SDD was also applied via a transanally placed Foley catheter (TAFC) for 48 h postoperatively. A propensity score-matched analysis was performed to identify risk factors for AL and SSI. Costs were calculated based on German diagnosis-related group (DRG) fees per case.

**Results:**

A total of 308 patients (154 per cohort) with a median age of 62.6 years (IQR 52.5–70.8) were analyzed. AL was observed in ten patients (6.5%) in the PC and 23 patients (14.9%) in the CC (OR 0.380, 95% CI 0.174–0.833; *P* = 0.016). SSI occurred in 14 patients (9.1%) in the PC and 30 patients in the CC (19.5%), representing a significant reduction in our SSI rate (*P* = 0.009). The cost-effectiveness analysis showed that SDD is highly effective in saving costs with a number needed to treat of 12 for AL and 10 for SSI.

**Conclusion:**

SDD significantly reduces the incidence of AL and SSI and saves costs for the general healthcare system.

**Supplementary Information:**

The online version contains supplementary material available at 10.1007/s00423-022-02540-6.

## Introduction

The field of colorectal surgery has experienced magnificent improvements over the last decades. As a result, colorectal cancer has become one of the domains of surgical oncology with outstanding chances of complete cure [1]. Furthermore, this progress has led to significantly better patient comfort, fewer Hartmann’s procedures, and shorter hospital stays. However, surgical site infection (SSI) and anastomotic leakage (AL), followed by local infection or sepsis, remain relevant, frequent, and severe postoperative complications, especially in rectal surgery. Reported rates of AL after low anterior resections for rectal cancer are still within a range of 10–20% and the negatively influence quality of life, oncological outcome, and costs [2].

The indication for modern preoperative mechanical bowel preparation (MBP) either alone or in combination with oral antibiotics (oral antibiotic bowel preparation [OABP]) to prevent the aforementioned complications has been a subject of ongoing controversy since its introduction in the 1970s [3, 4]. The use of bowel preparation before elective colorectal resection decreased in the beginning of the twenty-first century due to the failure of numerous clinical trials to demonstrate a positive effect against postoperative SSI or AL [5]. For some years, however, MBP and OABP have been experiencing a revival of interest as a result of studies showing their potential benefits in terms of reducing surgical site infection rates, anastomotic leakage, length of hospital stays, and readmission rates [6, 7]. Several of these studies have been done using large national databases of patients who underwent elective colorectal resections. However, most of them suffer from inconsistent protocols for both MBP and OABP. Furthermore, it is still unclear whether MBP, OABP, or a combination of both influences anastomotic healing. In addition, the pathophysiology of anastomotic leakage is still not fully understood. While the importance of blood perfusion and tensionless anastomosis is undisputed, the role of gut bacteria and the influence of topical anti-infective drugs remain largely unclear.

Recent studies have shown that selective decontamination of the digestive tract (SDD) using topical antimicrobial agents has a positive effect on the rate of anastomotic leakage. A reduction in anastomotic leakage could be demonstrated for both upper gastrointestinal tract (esophageal resections for cancer with esophageal-intestinal anastomosis) [8] and lower rectum resections [9]. In addition to the overall occurrence of AL, our goal was to clarify, if patients that experience an AL would have local or systemic infections and require reoperations less frequently.

Based on the promising evidence, perioperative SDD was implemented at our institution in September 2018 for all elective colorectal procedures. We aimed to determine the association between this change in preoperative procedure and occurrence of AL and SSI with regard to our postoperative management.

This report demonstrates the influence of MBP with SDD on the outcome of patients who received conventional or minimally invasive elective colorectal resection for benign and malignant diseases. In addition, a cost-effectiveness analysis was performed.

## Patients and methods

### Patients

This retrospective study was approved by the local institutional review board (decision number EK-347072021 and conducted in accordance with the Declaration of Helsinki and the ICH Harmonized Tripartite Guideline for Good Clinical Practice.

All patients who underwent elective colorectal operations between November 2016 and March 2020 were included in our analysis. Since our regimen of OABP and MBP started in September 2018, the analysis included two patient groups: a primary cohort (PC) comprising patients who received our regimen between September 2018 and March 2020 and a historic control cohort (CC) without standardized MBP operated between November 2016 and August 2018. Prior to the introduction of OABP and MBP, only patients with planned rectal resection received full MBP. Sigmoid and left-sided resection only had a transanally applied enema prior to surgery. Right-sided and transverse colonic resections did neither take OABP nor MBP. During the observation period, there was no change in suture material (PDS 4–0 or 5–0) or stapling device.

### Perioperative setting

Operations were performed or supervised by an experienced, certified colorectal surgeon. All patients were treated according to enhanced recovery after surgery (ERAS) principles [10]. Clinical data were retrospectively obtained from our internal documentation system.

All patients received perioperative intravenous antibiotics of cefuroxime 1.5 g and metronidazole 500 mg 30 min up to 1 h before surgery and repeated every 2 and a half hours after informed consent. In the case of allergy to cefuroxime, clindamycin 600 mg was given and repeated every 2 h. No postoperative intravenous antibiotics were routinely administered. The day before surgery, mechanical bowel preparation was performed using 3 l of solution for gastrointestinal lavage (polyethylene glycole-electolyte-based solution for gastrointestinal lavage, e.g., Klean-Prep®, Norgine GmbH, Marburg, Germany or Endofalk classic®, Dr. Falk Pharma GmbH, Freiburg, Germany). The ingredients are shown in Supplementary Table S2.

The SDD medication was prepared by our clinic pharmacy with a stability-approved protocol according to the pattern used in SELECT trial expanded by vancomycin [11, 12]. A combination of colistin base (100 mg), tobramycin base (80 mg), and amphotericin B (500 mg) and vancomycin (125 mg) was applied separately (10 ml each) due to stability concerns adding vancomycin to the formulation. Amphotericin B was given to prevent potential fungal superinfections, and vancomycin was added for the additional coverage of gram-positive germs. Patients received two oral doses of SDD and vancomycin the evening (4–8 p.m., after mechanical bowel preparation) before operation and one dose in the morning on the day of operation. Patients with an existing ileostomy had no mechanical bowel preparation. The SDD and vancomycin solutions were applied via the efferent loop of the ileostomy. In patients who underwent surgery of the rectosigmoid area, an unblocked 16F Foley catheter (fixed to the skin with sutures) was anally placed with the tip orally of the newly created anastomosis intraoperatively. A freshly prepared mixture of 10 ml SDD and 10 ml vancomycin solution diluted in 30 ml NaCl was administered via the catheter every 6 h starting intraoperatively after negative air pressure testing of the anastomosis. After application, the catheter was clamped for 1 h. The catheter was removed after 48 h. Signed informed consent for the off-label use of SDD for this indication was obtained from patients preoperatively.

In case of postoperative diarrhea, routine testing towards clostridium dificile toxin A/B was applied.

### Definition of postoperative complications

AL was defined according to the International Study Group of Rectal Cancer as a defect of the entire intestinal wall at the anastomotic suture line resulting in communication between the intra- and extraluminal spaces, detected by means of endoscopy, radiological contrast enema, thin layer CT, or surgery [13]. The complication of an abscess was either distant to the anastomosis or if it was in contact to the anastomosis it only counted as an abscess, if the criteria for AL were not fulfilled.

The Centers for Disease Control and Prevention define surgical site infection as infection that occurs at the incision site within 30 days after surgery, is limited to the cutis and subcutis, and requires reopening or treatment [14]. Additionally, patients with burst abdomen, no intra-abdominal infectious finding in reoperation, and clear evidence of infection (macroscopically plus intraoperative micobiological wound swap), were counted as SSI as well.

### Costs of bowel preparation and SDD

For mechanical bowel preparation, 3 l of coloscopy solution (Klean-Prep®) are used at a price of €3.53 per liter for a total of €10.59. The price of vancomycin is €13.53 for 1 vial of 500 mg, from which 125 mg is withdrawn. Patients without a TAFC require three doses (€10.15) and those receiving a TAFC need 11 (€37.21) doses of vancomycin. The cost of SDD suspension is €4829.20 for 100 bottles (containing 40 ml). One application contains 10 ml, so every patient without TAFC needs one bottle and patients with TAFC need three bottles. This finally comes to €58.44 for patients without a TAFC and €182.09 for those with a TAFC. In total, our regimen with bowel preparation and SDD solution costs €69.03 for patients without a TAFC and €192.68 for those with a TAFC.

### Costs of hospital stay

The standardized payments and weights of the German diagnosis-related group (G-DRG) system were used to calculate the total cost of hospital stay plus readmission.

### Statistics

Due to the retrospective character of the reported data, the sample size was not chosen based on a power calculation. Propensity scores for both the primary and control cohort were calculated with a multivariate logistic regression model including 14 variables (see Supplementary Table 1). Patients in the two cohorts were matched 1:1 with a difference between propensity scores of maximum 0.10. Continuous variables were expressed as median and interquartile range (IQR) and compared using Student’s *t* test or the Wilcoxon rank sum test. Dichotomous data were compared using the *χ*^*2*^ test*.* All variables with *P* < 0.1 were included in a stepwise backward multivariate logistic regression model adjusting for age, sex, BMI, and ASA. Results were reported as odds ratios (OR) and 95% confidence intervals (95% CI). No adjusting for multiple testing was applied. A *P* value < 0.05 was considered statistically significant. The number needed to treat was calculated as 1/absolute risk reduction. The absolute risk reduction was the difference between the event rates in the PC and CC. Statistical analyses were carried out using IBM SPSS Statistics v23 (IBM Corp., Armonk, NY).

## Results

### Patient characteristics

A total of 308 patients with 154 patients in each group (PC and CC) were selected via propensity score analysis. The median age was 63.1 (IQR 54.8–70.8) years in the PC and 61.9 (IQR 51.9–71.9) years in the CC. Body mass index (BMI) was the same in both the PC (25.5; IQR 22.8–29.1) and CC (25.5; IQR 22.6–29.0). The two cohorts did not differ in most of the investigated base line parameters. However, there was a significant difference in terms of previous abdominal operations, including gall bladder and hernia operations. Patients in the PC had previous surgery in 66/154 (42.9%) cases, whereas those in the CC received previous abdominal surgery in 88/154 (57.1%) cases (*P* = 0.012). The main indication for surgery in both cohorts was a tumor in 116/154 (75.3%) and 114/154 (74.0%) patients in the PC and CC, respectively. This was followed by inflammatory bowel disease (Crohn’s disease, ulcerative colitis, diverticulitis, and others) in 21/154 (13.6%) patients in the PC and 22/154 (14.3%) in the CC. Reconstruction after Hartmann’s procedure was performed in 11/154 (7.1%) patients in the PC and 12/154 (7.8%) patients in the CC (Table 1).

**Table 1:** Patients’ characteristics and preoperative data

	Control cohort without SDD *n* = 154		Primary cohort with SDD *n* = 154		
	No. (%)	Median (IQR)	No. (%)	Median (IQR)	*P* value
Age	154	61.9 (51.9–71.9)	154	63.1 (54.8–70.8)	0.868
Gender					0.906
Male	96 (62.3)		97 (63.0)		
Female	58 (37.7)		57 (37.0)		
BMI	154	25.5 (22.8–29.1)	154	25.5 (22.6–29.0)	0.555
ASA					0.305
I	17 (11.0)		17 (11.0)		
II	65 (42.2)		57 (37.0)		
III	71 (46.1)		77 (50.0)		
IV	1 (0.6)		3 (1.9)		
Previous operations					**0.012**
No	66 (42.9)		88 (57.1)		
Yes	88 (57.1)		66 (42.9)		
Indication					
Tumor (incl. hereditary and benign)	114 (74.0)		116 (75.3)		
Ulcerative colitis	4 (2.6)		4 (2.6)		
Crohn’s disease	9 (5.8)		11 (7.1)		
Diverticulitis	9 (5.8)		7 (4.5)		
C.a. Hartmann	12 (7.8)		11 (7.1)		
Consequence of other treatment	4 (2.6)		4 (2.6)		
Others (stenosis, fistula)	2 (1.3)		1 (0.6)		
Type of disease					0.902
CRC	88 (57.1)		86 (55.8)		
Other malignancies	18 (11.7)		20 (12.9)		
Benign	48 (31.2)		48 (31.2)		
Neoadjuvant radiotherapy					0.692
No	139 (90.3)		141 (91.6)		
Yes	15 (9.7)		13 (8.4)		
Albumin preoperative [g/l]	150	43.4 (39.6–46.1)	146	44.2 (40.9–46.5)	0.317

Significant *P*-values are presented in bold

*SDD*, selective digestive decontamination; *No*., number of patients; *IQR*, interquartile range; *BMI*, body mass index; *ASA*-score, American Society of Anesthesiology score; *C.a*., condition after; *CRC*, colorectal cancer; *g/l*, gram per liter

Surgical interventions were evenly balanced between the cohorts. In summary, 103 (66.9%) patients in the PC and 107 (69.5%) in the CC underwent colonic surgery, and 51 (33.1%) patients in the PC and 47 (30.5%) in the CC had rectal resections. The most common operations were right hemicolectomy with 42 operations in the PC (27.2%) and 34 (22.1%) in the CC, followed by rectal and sigmoid resections in 19 (12.3%) patients in the PC and 25 (16.2%) in the CC. Multivisceral resection was necessary (> 2 organs, parts of the bowel) in 20 (13.0%) patients in the PC and 24 (14.3%) in the CC. Two anastomoses were performed on nine patients in the PC (5.8%) and six patients in the CC (3.9%). Three anastomoses to the colon were carried out in two patients in the CC (1.3%) and 0 patients in the PC. A minimally invasive approach was used in 80 patients in the PC (51.9%) and 74 patients in the CC (48.1%). One hundred sixteen (75.3%) patients in the PC and 114 (74.0%) in the CC have been operated due to a tumor (Table 2).

**Table 2:** Intra- and postoperative outcome

	Control cohort without SDD *n* = 154		Primary cohort with SDD *n* = 154		
	No. (%)	Median (IQR)	No. (%)	Median (IQR)	*P* value
Operating time (min)	154	281.0 (191.8–395.5)	154	312.5 (227.8–412.0)	0.067
Hospital stay (d)	154	11.5 (8.0–20.0)	154	9.5 (8.0–14.3)	0.137
Operational procedures					
Ileocecal resection	10 (6.5)		15 (9.7)		
Hemicolectomy right	42 (27.2)		34 (22.1)		
Hemicolectomy left	8 (5.2)		8 (5.2)		
Sigmoid resection	19 (12.3)		25 (16.2)		
Rectal resection	49 (31.8)		51 (33.1)		
Colonic segmental resection	3 (1.9)		1 (0.6)		
Colectomy	6 (3.9)		6 (3.9)		
Reconstruction Hartmann	12 (7.8)		11 (7.1)		
Others	5 (3.2)		3 (1.9)		
Stool diversion					0.084
No	123 (79.9)		110 (71.4)		
Yes	31 (20.1)		44 (28.6)		
Invasiveness					0.792
Open	67 (43.5)		64 (41.6)		
Laparoscopic	40 (26.0)		33 (21.4)		
Robotic assisted	34 (22.1)		45 (29.2)		
Laparoscopic conversion	10 (6.5)		9 (5.8)		
Robotic conversion	3 (1.9)		3 (1.9)		
Mortality (30 d)					0.317
No	153 (99.4)		154		
Yes	1 (0.6)		0		
Morbidity (incl. SSI)					0.133
No	103 (66.9)		115 (74.7)		
Yes	51 (33.1)		39 (25.3)		
Clavien-Dindo score					0.413
0–2	118 (76.6)		132 (85.7)		
3–5	36 (23.4)		22 (14.3)		
Surgical site infection (SSI)					**0.009**
No	124 (80.5)		140 (90.9)		
Yes	30 (19.5)		14 (9.1)		
Major surgical complication*					**0.050**
No	110 (71.4)		123 (79.9)		
Yes	44 (28.6)		31 (20.1)		
Surgical complication detail					
Anastomotic leakage	23 (14.9)		10 (6.5)		**0.017**
Postoperative bleeding	6 (3.9)		5 (3.2)		
Abscess	12 (7.8)		6 (3.9)		
Burst abdomen	6 (3.9)		3 (1.9)		
Post-op antibiotics-associated colitis	2 (1.3)		2 (1.3)		
Others (stoma, fistula, ileus, etc.)	15 (9.7)		16 (10.4)		
Re-surgery					0.092
No	123 (79.9)		134 (87.0)		
Yes	31 (20.1)		20 (13.0)		

Significant *P*-values are presented in bold

*SDD*, selective digestive decontamination; *No*., number of patients; *IQR*, interquartile range; *BMI*, body mass index; *min*, minutes; *d*, days; *SSI*, surgical site infection; *g/l*, gram per liter

^*^except SSI

### Morbidity and mortality

Postoperative morbidity was 25.3% in the PC and 33.1% in the CC (*P* = 0.133). Additionally, the Clavien-Dindo score is reported (Table 2).

AL occurred in ten patients (6.5%) in the PC and 23 patients (14.9%) in the CC, which represented a significant reduction (*P* = 0.017). In our CC, 31 patients (20.1%) required redo surgery, whereas in the PC, only 20 patients (13.0%) needed surgical reintervention. The reason for reoperation was mainly due to AL (PC: 9; CC: 16), but also four patients with postoperative bleeding in CC. In total, SSI (PC: 4; CC: 3) and burst abdomen due to SSI (PC: 2; CC: 3) were the main reasons for repeated surgery (Table S4). This reduction showed a trend towards significance (*P* = 0.092).

In PC, the nine patients with reoperation due to AL were six Hartmann’s procedures, two local revision, and one new creation of the anastomosis. In CC, AL reoperation was as follows: ten patients with Hartmann’s procedure, five local revision of the anastomosis (two patients in need for extra protective loop ileostomy), and one newly generated anastomosis.

SSI occurred in 30 patients in the CC (19.5%) and 14 patients (9.1%) in the PC, which resulted in a significant reduction in our SSI rate (*P* = 0.009).

The individual surgeon’s qualification (certified or not) had no influence on postoperative outcome, neither for AL (*P* = 0.101) nor for SSI (*P* = 0.843). Subgroup analysis also showed no influence on AL and SSI for malignant/benign and inflammatory vs. not inflammatory disease.

Hospital readmission occurred in 6/275 (2.2%) patients without AL and in 13/33 (39.4%) patients with AL (*P* < 0.001). Of the patients with SSI, 5 out of 44 (11.4%) were readmitted to the hospital compared to 14/264 patients (5.3%) without SSI (*P* = 0.122).

Additional interventions due to anastomotic leakage were done by CT-guided drain placement in four patients in the PC and ten patients in the CC. One patient in the PC and two patients in the CC had endorectal vacuum therapy for AL.

Three patients in the CC died during the hospital stay, resulting in an in-hospital mortality rate of 1.9% in the CC and 0% in the PC. The 30-day mortality rate was 0.6% (one patient) in the CC and 0% in the PC.

### Risk factors for anastomotic leakage

Uni- and multivariate analysis revealed SDD as the only independent factor to associate with a reduced rate of AL (univariate: *P* = 0.017; multivariate: OR 0.380, 95% CI 0.174–0.833; *P* = 0.016) (Table 3).

**Table 3:** Analysis towards anastomotic leakage

	Anastomotic leakage					
			Univariate	Multivariate		
	No (mean)	Yes (mean)	*P* value	OR	95% CI	*P* value
Age	60.5	61.1	0.788			0.753
Gender			0.615			0.537
Male	171	22				
Female	104	11				
BMI	26.1	27.2	0.154			0.248
ASA			0.184			0.141
I/II	142	13				
III/IV	133	20				
Previous operations			0.580			
No	136	18				
Yes	139	15				
Operating time (min)	320.7	353.8	0.191			
Height of operation			0.166			
Colon	191	19				
Rectum	84	14				
Invasiveness			0.429			
Open	112	17				
Minimally invasive	141	13				
Conversion	22	3				
Type of disease			0.876			
Benign	87	10				
Malign	188	23				
Neoadj. radiotherapy			1.000			
No	250	30				
Yes	25	3				
Albumin preop g/l	42.7	40.3	0.161			
Stool diversion			0.382			
No	206	27				
Yes	69	6				
SDD			**0.017**	**0.380**	**0.174–0.833**	**0.016**
No	131	23				
Yes	144	10				

Significant *P*-values are presented in bold

*AL*, anastomotic leakage; *OR*, odds ratio; *95% CI*; 95% confidence interval; *BMI*, body mass index; *ASA*-s*core*, American Society of Anesthesiology score; *min*, minutes; *g/l*, gram per liter; *SDD*, selective digestive decontamination

### Risk factors for surgical site infection

In univariate analysis, higher ASA scores of 3 or 4 (*P* = 0.020), previous operations (*P* = 0.003), and other surgical complications (*P* < 0.001) were associated with higher SSI rates. Patients with higher preoperative albumin (*P* = 0.002) and stool diversion (*P* = 0.011), who had undergone minimally invasive (laparoscopic or robotic) (*P* < 0.001) surgery or received SDD, had a significantly lower incidence of SSI. Multivariate analysis found minimally invasive operations (OR 0.273, 95% CI 0.113–0.658; *P* = 0.004) and high albumin levels (OR 0.915, 95% CI 0.867–0.965; *P* = 0.001) to be associated with a reduced rate of SSI. An additional surgical complication was the strongest predictor of SSI (OR 6.058, 95% CI 2.809–13.069; *P* < 0.001). SDD just failed to reach a level of significance in multivariate analysis (*P* = 0.057). The results are summarized in Table 4.

**Table 4:** Analysis towards surgical site infection

	Surgical site infection					
			Univariate	Multivariate		
	No (mean)	Yes (mean)	*P* value	OR	95% CI	*P* value
Age	60.4	61.4	0.665			0.864
Gender			0.248			0.054
Male	162	31				
Female	102	13				
BMI	26.2	26.3	0.758			0.621
ASA			**0.020**			0.456
I/II	140	15				
III/IV	124	29				
Previous operations			**0.003**			0.267
No	141	13				
Yes	123	31				
Operating time (min)	329.8	290.7	0.206			
Height of operation			0.080			
Colon	175	35				
Rectum	89	9				
Invasiveness			** < 0.001**			
Open	96	33				
Minimally invasive	145	9		**0.273**	**0.113–0.658**	**0.004**
Conversion	23	2				
Type of disease			0.271			
Benign	80	17				
Malign	184	27				
Neoadj. radiotherapy			0.571			
No	239	41				
Yes	25	3				
Surgical complication			** < 0.001**	**6.058**	**2.809–13.069**	** < 0.001**
No	214	16				
Yes	50	28				
Albumin preop g/l	43.3	37.7	**0.002**	**0.915**	**0.867–0.965**	**0.001**
Stool diversion			**0.011**			0.086
No	193	40				
Yes	71	4				
SDD			**0.009**			0.057
No	124	30				
Yes	140	14				

Significant *P*-values are presented in bold

*SSI*, surgical site infection; *OR*, odds ratio; *95% CI*; 95% confidence interval; *BMI*, body mass index; *ASA*-*score*, American Society of Anesthesiology score; *min*, minutes; *g/l*, gram per liter; *SDD*, selective digestive decontamination

### Effect of SDD via transanally placed Foley catheter (TAFC)

In total, 83 patients (53.9%) in the PC received postoperative SDD via a catheter placed over the rectal anastomosis. Seventy-five patients (48.7%) in CC are the equivalent patient cohort with rectal anastomosis to perform a subgroup analysis. In total, 8/83 (9.6%) patients in the PC and 12/75 (16.0%) in the CC developed an AL. Univariate analysis did not reach the level of significance (*P* = 0.230).

In patients with a diverting stoma, 2 out of 44 (4.5%) in the PC and 4 out of 31 (12.9%) in the CC were found to have an AL, which was not statistically significant (*P* = 0.189).

There was no significant detectable influence of SDD on SSI in the subgroup of patients with a transanally placed catheter (*P* = 0.144) or diverting stoma (*P* = 0.160). Also no complication or anaphylactic reaction was seen due to catheter placement and SDD application.

### Costs of AL

All 308 patients were in the hospital for 4897 days with a median of 11.5 days (IQR 8.0–20.0), resulting in costs of €4,760,461 for the healthcare system. The median DRG relative weight was 3.55 (IQR 2.8–4.5). Patients without AL were hospitalized for a median of 9.0 days (IQR 8.0–14.0) and those with AL for 28.0 days (IQR 19.5–48.0). Overall, this came to 627 additional hospital days for the AL group. The median hospital merit for one patient with AL corresponds to a DRG weight of 6.25 (IQR 4.6–10.5) at costs of €19,081.00 (IQR 15,585.50–32,641.00) compared to a DRG weight of 3.41 (IQR 2.8–4.1) at costs of €11,850.00 (IQR 9333.00–14,082.00) for non-AL patients. On average, our hospital calculated an additional €7231.00 for every patient with AL. These patients were hospitalized for a median of 19.0 additional days.

Patients in the CC did not receive SDD, were hospitalized for a median of 11.5 days (IQR 8.0–20.0), and generated a median DRG weight of 3.55 (IQR 2.8–4.6), resulting in median revenue for the hospital of €12,209.00 (IQR 9333.00–15,163.75) per patient. In contrast, the introduction of SDD resulted in a median hospital stay of 9.5 days (IQR 8.0–14.3), a median DRG weight of 3.46 (IQR 2.8–4.0), and the same median revenue of €12,209.00 (IQR 9931.30–14,160.00). In summary, in our SDD group, we saved a median of 2.0 hospital days (a total of 308 hospital days) at moderate treatment costs for the procedure and medication (€4832.10 + €15,992.44 = €20,824.54).

With regard to AL, we had 8.5% fewer insufficiencies (PC: 10/154 vs. CC: 23/154) since the introduction of SDD. As expected, AL corresponds to a median DRG weight increase of 2.84. This resulted in extra expenses of approximately €135,000 in our cohort (based on a basic case value of €3680 in Germany in 2020), which might have been prevented with the use of SDD.

### Costs of SSI

**Figure1:**
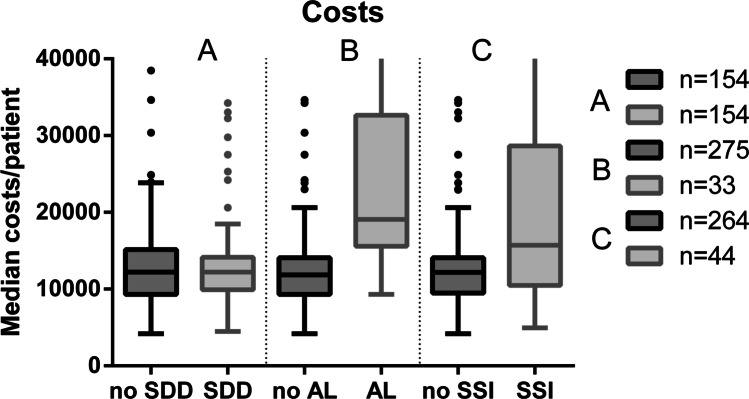
Comparison of treatment costs. Median treatment costs in € between patients receiving SDD or not (**A**), patients with AL or not (**B**), and patients with SSI or not (C) are demonstrated. AL, anastomotic leakage; SDD, selective decontamination of the digestive tract; SSI, surgical site infection; n, number

We calculated the costs associated with SSI as follows: Patients in the CC without SSI had a median hospital stay of 10.0 days (IQR 8.0–15.8), corresponding to a DRG weight of 3.49 (IQR 2.8–4.2) and median costs of €12,029.50 (IQR €9307.00–€14,362.00). In comparison, patients in the CC with SSI (*n* = 30) had a median stay of 26.0 days, corresponding to a median DRG weight of 4.71 (IQR 3.1–8.9) and median costs of €15,649.00 (IQR €11,357.75–25,567.00). Compared to CC data (30 patients with SSI), the regular use of SDD led to 14 patients with SSI in PC, thus 16 patients less suffered SSI. Taking into account the longer hospital stay of 16 days (IQR 10.0–26.0 days) and higher costs of €3620.00 (calculated for the CC) per patient, SDD led to a cost reduction of €57,920.00 (16 × €3620.00) and we saved 256 hospital days (16 × 16) in our PC. When assuming the costs for SSI, a notification towards a strong bias of other complications (e.g., AL) potentially triggering the presence of SSI, needs to be mentioned (Fig. 1).

### Number needed to treat (NNT)

Finally, we calculated the number needed to treat. To do this, the absolute risk reduction rate was determined by subtracting the experimental event rate from the control event rate. This resulted in a NNT of 11.8; therefore, 12 patients need to be treated with SDD in order to prevent 1 AL. Using the same calculation for SSI resulted in a NNT of 9.6, meaning that ten patients need to be treated with SSD to prevent 1 SSI. Looking at the costs for SDD, the amount that needs to be invested to prevent AL in one single patient ranges from €828.36 to €2312.16, depending on whether a TAFC is used, €69.03 for patients without a TAFC, and €192.68 for those with a TAFC.

## Discussion

In our study, we demonstrated that SDD in combination with MBP has a high potential to reduce AL and SSI. In addition, we provide a cost-effectiveness calculation which might help to spread the use of SDD in routine clinical practice. In summary, we show that using SDD can help reduce costs significantly. Our results confirm the findings of previous studies on this topic [15–17]. Our SDD regimen is quite similar to the one used in the SELECT trial, and confirms the results of the trial, resulting in a high potential to reduce infectious surgical complications [12]. There has been ongoing debate about whether patients should receive antibiotics or MBP alone or in combination before bowel operation. Many convincing studies suggest that MBP alone is not effective in improving postoperative outcomes and may also harm the patient. It should therefore be administered in combination with antibiotics [18–21]. Nevertheless, from a surgical point of view, MBP is preferable in terms of cleaner and easier handling during and after operational procedures. In addition, small tumors can be better identified in open or laparoscopic surgery. Furthermore, non-palpable tumors or polyps are easier to detect by endoscopy while surgery is in progress. Recently, a meta-analysis including 57,207 patients found oral antibiotics in combination with MBP to be superior to MBP alone in reducing SSI [22]. The argument that SDD causes more antibiotic-related *Clostridium difficile* (CD) infections could not be confirmed in our cohort (equal distribution) due to the additional use of vancomycin. Moreover, it has been reported to decrease rather than increase the number of CD infections after colectomy [23]. This risk only seems to be elevated in patients with pre-existing systemic inflammatory response syndrome [24]. What still remains unsolved, is the question about compound and dosing of the antibiotics. The use of MBP in combination with anti-infectives reduces the bacterial load of both Proteobacteria and *Enterobacteriaceae* and fungi in the colon by using effective broad-spectrum antibiotic agents (colistin and tobramycin) against aerobic and anaerobic bacteria, vancomycin against gram-positive bacteria (especially enterococci species), and amphotericin B against fungi. In particular, bacteria species that produce enzymes which disturb anastomotic or wound healing (proteases, collagenases), like *Enterococcus* *faecalis*, *Pseudomonas aeruginosa*, *and Serratia marcescens*, are effectively reduced. These potentially pathogenic microorganisms are thus prevented from overproducing and disrupting the balance of the gut microbiome [12, 19, 25]. Topical antibiotics used in combination with intraoperatively administered intravenous antibiotics effectively suppress mucosal-associated flora in the colon and, thus, prevent the surgical wounds of contamination [26].

In 2018, nearly 79.3% of surgeons in the USA used oral antibiotics in combination with MBP. The most widely used oral antibiotic regimen was neomycin and metronidazole. In a European survey (2018), only 16.8% of patients with elective left-sided colon and rectal resection received a combination of MBP and oral antibiotics. These numbers highlight the need to establish SDD and MBP more firmly in routine clinical practice [27–29]. With respect to the literature, it remains unclear whether patients benefit from topical use via a Foley catheter. Regarding our results in the TAFC group, a clear trend towards improvement of AL and SSI could also be seen in the group with a diverting stoma. However, our patient cohort might be too small to give a precise recommendation for applying SDD via transanally placed Foley catheters. More randomized trials and experience are needed to address this matter [9]. Furthermore, the question of which medication to use and at what dosage is a topic to be addressed in future trials. Our regimen seems to be highly effective considering current knowledge, but the wide field of the gut microbiome still remains hardly understood with more intensified research needed [25].

## Limitations

We are aware that our study has several weaknesses. The effect of MBP alone cannot be illustrated due to the retrospective character of our data and the different use of MBP in our CC. By using propensity score matching, we tried to generate homogeneous patient cohorts and control for bias. Nevertheless, our data is unicentric and not comparable to that of large multicenter randomized trials in terms of number of patients. However, it represents the average patients of a German university hospital, taking into consideration the need for surgical education, the high numbers of pre-existing illnesses at risk in the patient cohort, and more extensive surgical procedures, often in patients with previous abdominal surgeries.

With regard to our cost analysis, we calculated the maximum amount per single application of SDD based on the official pharmaceutical catalog prices. In practice, the drug prizes for hospitals may be lower, and the costs for SDD and MBP may be less than in our calculation. For the SSI calculations, indirect costs (additional costs for regular visits, wound dressing materials, and specialized wound nurses) could not be considered due to lack of data. By using the DRG system to calculate the costs for the healthcare system, only the reimbursement volumes are measured. This might be different to the real costs of the complications, which cannot be measured easily without direct data of medical insurance companies. As indirect costs are difficult to calculate, there is a wide range of costs reported. Since costs in the ambulatory setting are frequently not considered, we focused on hospital costs. With regard to previous reports, a median of €5000 in additional costs can be assumed for every patient with SSI [30–33]. In our cost-effectiveness analysis, we could demonstrate that savings of over 300 hospital days in a relatively small patient cohort (*n* = 154) can be achieved by using our SDD regimen. Although patients suffering from AL led to higher revenue of over €7000 per patient, in-hospital costs for AL treatment (besides the much longer stay) cannot be calculated and are likely much higher. In short, many patients can be spared the consequences of AL and SSI for little investment and an acceptable NNT of 12 and 10, respectively. We therefore strongly recommend including SDD in the existing bundle of measures to prevent surgical side effects [34].

## Conclusion

SDD in combination with mechanical bowel preparation is a highly effective procedure to reduce the risk of AL and SSI in patients requiring elective colorectal resection and restoration. Due to the high increase in costs and significantly longer hospital stay for patients with AL and SSI after surgery, we highly recommend the standard use of an effective combination of antibiotic and antimycotic drugs (in our setting, this includes colistin, tobramycin, vancomycin, and amphotericin B) before any elective colorectal operation. In our propensity score-matched cohort of 308 patients, we could demonstrate a remarkable cost-effectiveness analysis, which revealed a high potential for reducing costs for the hospital and healthcare system by shortening the length of hospital stay and reducing complications at moderate treatment costs. More importantly, however, is the improved outcome for patients in terms of hospitalization, immobility, pain, and sick leave. The use of topical SDD via a Foley catheter after colorectal anastomosis is still controversial, and the demonstrated trend in patients receiving diverting stomas towards a reduction of AL may be a topic of further studies.

## Supplementary Information

Below is the link to the electronic supplementary material.

Supplementary file1 (DOCX 17 KB)

## Data Availability

All authors declare that they had full access to all information, data, software, and codes used for and published in this article. These data have not been published elsewhere.
