# Supplementary material for: Selective decontamination of the digestive tract in colorectal surgery reduces anastomotic leakage and costs: a propensity score analysis
Source: Langenbecks Arch Surg. 2022 May 13;407(6):2441–52. doi: 10.1007/s00423-022-02540-6 (PMC9468075; doi:10.1007/s00423-022-02540-6)
Supplement: Supplementary file 1 — Supplementary file1 (DOCX 17 KB) [file 423_2022_2540_MOESM1_ESM.docx]

**Supplementary data:**

**Table S1: List of all 14 variables for propensity scoring**

1. Age

2. Gender (male/female)

3. Type of operational procedure

4. Category

5. Indication

6. Colonic vs. Rectal resection

7. Minimally invasive (yes/no)

8. Type of disease

9. Preoperative radiation (yes/no)

10. ASA (I/II vs. III/IV)

11. BMI (cut off 25.0 kg/m²)

12. Albumine (cut off: 3.5 g/dl)

13. Previous abdominal operation (yes/no)

14. Stool diversion (yes/no)

**Table S2: Ingredients of KLEAN-PREP®**

| **1 bag (69 g powder) contains** | **69g dissolved in 1 litre of water** |
| --- | --- |
| Macrogol 3350 59.000 g | Sodium 125.0 mmol/l |
| Natriumsulfat 5.685 g | Potassium 10.0 mmol/l |
| Natriumhydrogencarbonat 1.685 g | Sulfat 40.0 mmol/l |
| Natriumchlorid 1.465 g | Chloride 35.0 mmol/l |
| Kaliumchlorid 0.743 g | Hydrogencarbonat 20.0 mmol/l |
|  | Macrogol 3350 17.6 mmol/l |

Ingredients of Endofalk classic®

| **1 bag (55 g powder) contains** | **110g (2 bags) dissolved in 1 litre of water** |
| --- | --- |
| Macrogol 3350 52.500 g | Sodium 65.0 mmol/l |
| Natriumhydrogencarbonat 0.715 g | Potassium 5.0mmol/l |
| Natriumchlorid 1.400 g | Chloride 53.0 mmol/l |
| Kaliumchlorid 0.185 g | Hydrogencarbonat 17.0 mmol/l |
|  | Macrogol 6700 35.2 mmol/l |

Table S3: ERAS criteria according to Gustafsson et al. (World J Surg 2019)

| ERAS Item | In clinical use |
| --- | --- |
| 1) Preadmission information, education and counselling | Yes |
| 2) Preoperative optimisation | In parts |
| 3) Prehabilitation | Yes |
| 4) Preoperative nutritional care | Yes |
| 5) Management of Anaemia | Yes |
| 6) Prevention of nausea and vomitiong (PONV) | Yes |
| 7) Pre-anaesthetic medication | Yes |
| 8) Antimicrobial prophylaxis and skin preparation | Yes |
| 9) Bowel preparation | Yes in PC, only rectal resections in CC |
| 10) Preoperative fluid and electrolyte therapy |  |
| 11) Preoperative fasting and carbohydrate loading | No |
| 12) Standard Anaesthetic Protocol | Yes |
| 13) Intraoperative fluid and electrolyte therapy | Yes |
| 14) Preventing intraoperative hypothermia | Yes |
| 15) Surgical access (open and minimally invasive surgery including laparoscopic, robotic and trans-anal approaches | Yes |
| 16) Drainage of the pertoneal cavity and pelvis | Yes |
| 17) Nasogastric intubation | Yes |
| 18) Postoperative analgesia | Yes |
| 19) Thromboprophylaxis | Yes |
| 20) Postoperative fluid and electrolyte therapy | Yes |
| 21. Urinary drainage | Yes |
| 22) Prevention of postoperative ileus | Yes |
| 23) Postoperative glycaemic control | Yes |
| 24) Postoperative nutritional care | Yes |

Table S4 Reasons for reoperation

| **Primary cohort n=20** | **Control cohort n=31** |
| --- | --- |
| Anastomotic leakage 9 | Anastomotic leakage 16 |
| SSI 4 | Postoperative bleeding 4 |
| Burst abdomen 2 | SSI 3 |
| Ileus due to narrow stoma 1 | Burst abdomen 3 |
| Paralytic ileus 1 | Ileus due to narrow stoma 1 |
| Adhesive ileus 1 | Paralytic ileus 1 |
| Colonic leason other than AL 1 | Abszess 1 |
| Pleura empyema 1 | Colonic leason other than AL 1 |
|  | Others (SMV occlusion) 1 |

Operative therapy AL in primary cohort:

Hartmann’s procedure: 6

Local revision of anastomosis: 2

New anastomosis: 1

Operative therapy AL in control cohort:

Hartmann’s procedure: 10

Local revision of anastomosis: 5 (2x protective ileostoma, 1x EndoVAC)

New anastomosis: 1
